# Unveiling the Biography of Sigmund Freud With Pediatric Dentistry Insights

**DOI:** 10.7759/cureus.69863

**Published:** 2024-09-21

**Authors:** Nilima R Thosar, Mrunali Deshkar, Monika Khubchandani, Rashi Dubey, Vipin Ahuja

**Affiliations:** 1 Pediatric and Preventive Dentistry, Sharad Pawar Dental College, Datta Meghe Institute of Higher Education and Research, Wardha, IND; 2 Pediatric and Preventive Dentistry, Government Dental College and Hospital, Jamnagar, IND

**Keywords:** historical vignette, human mind, psychoanalysis, theories, unconscious mind

## Abstract

Born in Freiberg, Moravia (now the Czech Republic), Austrian psychiatrist Sigmund Freud was the forerunner of psychoanalysis, a ground-breaking approach to understanding the human mind. Originally studying neurology at the University of Vienna, Freud soon became fascinated with the psychological origins of mental illness. He developed theories on repression, the unconscious mind, and the significance of dreams because he saw dreams as windows into hidden conflicts and impulses. Freud developed the theory and asserted that unconscious impulses, especially those related to sexuality, have a major impact on human conduct. Dream analysis and free association were among his innovative methods that helped modern psychotherapy come to pass. Though they were attacked and divisive, Freud's theories had a major impact on humanities, psychology, and psychiatry. His studies on the human mind challenged conventional wisdom and offered innovative insights into mental health. Freud's writings continue to inspire research and debate; his effect on popular and scholarly opinion attests to his ongoing legacy.

## Introduction and background

Sigmund Freud was born on May 6, 1856, in Freiberg, Moravia. He is regarded as one of the most influential psychiatrists of the 20th century. As the father of psychoanalysis, he brought forth revolutionary concepts that fundamentally altered the way society understood psychological processes in humans [1]. His studies on the subconscious mind, the mechanism of repression, and the significance of dreams broke new ground in psychology since they provided an in-depth understanding of human behavior, motivations, and development [2]. For instance, Freud introduced the groundbreaking concepts of the id, ego, and superego as well as the Oedipus and Electra complex to psychology. These concepts have had a significant impact on a wide range of fields, including literature, art, and cultural studies. His contributions were indispensable in the face of harsh criticism, making him a pivotal figure in the study of the human mind and experience [3].

Early life and education

Freud was the first of eight children born to Jakob Freud, a wool merchant, and Amalia Nathansohn, who was 20 years younger than her husband. Freud’s family was Jewish, and though his upbringing was modest, his parents were determined to provide him with a solid education [4]. In 1860, when Freud was four years old, the family moved to Vienna, where he would spend most of his life. Freud was an exceptionally bright student, showing an early aptitude for languages and a keen interest in literature and science. He attended the Leopoldstädter Kommunal-Realgymnasium, where he excelled academically, particularly in subjects like Latin, Greek, history, and mathematics. His intellect and ambition earned him a place at the University of Vienna in 1873, where he initially considered studying law but chose to pursue medicine instead [5].

At the university, Freud became deeply interested in biology and physiology, particularly the work of Charles Darwin, whose theories on evolution had a significant influence on his thinking. Freud worked under the renowned physiologist Ernst Brücke, who inspired him to delve into neuroanatomy. He graduated in 1881 with a medical degree, having specialized in neurology. Freud's early education laid the foundation for his later work in psychoanalysis, providing him with a solid grounding in the scientific methods and theories that would shape his groundbreaking explorations of the human mind [6].

## Review

Theoretical contributions and legacy

The Oedipus complex theory, one of Freud's most well-known theories, holds that kids harbor unconscious feelings of jealousy against their same-sex parent and unconscious sexual cravings for their opposite-sex parent. Both this idea and Freud's focus on the significance of sexuality in human development were very contentious at the time and are still up for discussion today [7,8]. Even though Freud's theories were groundbreaking, there was opposition to and mistrust of them. During his time, the conservative society viewed his emphasis on sexuality in particular as scandalous. Freud published a number of books, such as "The Interpretation of Dreams" (1900), "Three Essays on the Theory of Sexuality" (1905), and "Civilization and Its Discontents" (1930), in spite of this, and he continued to expand his views. The long-lasting influences of these works on psychology, literature, and the arts finally turned into cornerstones of psychoanalytic theory [9,10]. Apart from his intellectual achievements, Freud's works had a major influence on psychotherapy techniques. Therapists still use their innovative work on free association, dream analysis, and transference. Freud's ideas spawned other schools of psychology, including existential and humanistic psychology as well as the theories of Carl Jung and Alfred Adler [11]. Along with increasing our understanding of the human mind, Sigmund Freud made major contributions to the fields of art, literature, social theory, and psychology. Freud, the founder of psychoanalysis, presented novel ideas on sexuality, the unconscious, and personal behavior development. Because of the influence his work has had and will continue to have, he is among the most significant figures of the 20th century [11].

Psychoanalysis: a new approach to the mind

The most significant contribution Freud made was psychoanalysis, a method for treating mental illness and understanding human behavior [12]. Psychoanalysis stresses the unconscious mind, a repository of memories, impulses, and ideas that often impact our behavior without our conscious awareness [13,14]. Using free association, dream interpretation, and the study of linguistic errors is often referred to as "Freudian slips." Freud’s technique revealed these underlying energies. This approach transformed the treatment of mental health diseases and became the foundation for many of the therapeutic approaches still applied today [15].

The unconscious mind

Before Freud, most people agreed that unconscious influence had little bearing on human behavior. Freud first proposed that the unconscious is a strong force shaping our emotions, concepts, and actions. According to him, a lot of our mental life stays secret from conscious awareness, and unresolved conflicts and wants in the unconscious could lead to mental diseases. This thesis challenged the prevailing notion that the mind is a conscious, rational mechanism and offered novel angles on human motivation and behavior [16].

Theory of psychosexual development

The foundation of Freud's work is also his theory of psychosexual development. His hypothesis holds that during different phases of human growth, sexual energy, that is, desire, is directed to different physiological locations. Some issues relating to the oral, anal, phallic, latent, and genital stages have to be settled if one wants to grow psychologically in a healthy manner. Developmental psychology and education have been much influenced by Freud's ground-breaking attention on how early events build personality [17].

Defense mechanisms

First put up by Freud was the concept of defense mechanisms, psychological strategies the ego uses to protect itself from conflict and discomfort. Among other techniques, these ones comprise rationalizing, projecting, denial, and suppression. Freud's identification of these processes helps us to better grasp how individuals control stress and inner conflicts, which is advantageous for daily living as well as therapeutic activity [14].

Impact on culture and the arts

Beyond psychology, Freud's influence is seen in more general spheres of society, literature, and the arts. His ideas on sexuality, dreams, and the unconscious have shaped many writers, artists, and filmmakers. Ideas like the "Freudian slip" and dream interpretation have become rather common in popular culture. One method in which Freud's writings have shaped literary criticism is through the field of psychoanalytic criticism, which studies the psychological elements of works [18].

Challenging social norms

Furthermore, Freud was a social critic who challenged the accepted wisdom and taboos of the day, particularly with regard to sexuality. Though first controversial, his hypothesis that human sexuality is the main driver of life has now been generally embraced. By de-stigmatizing talks about sexuality and mental health, Freud's writings helped to advance open and progressive ideas in both spheres [19].

Freud’s theories on childhood development and the oral stage

One of Freud's major contributions to psychology was his theory of psychosexual development, which emphasizes the role of early childhood experiences in shaping an individual’s personality and behavior. According to Freud, the first stage of this development, known as the oral stage, occurs from birth to approximately 18 months. During this period, the mouth becomes the primary source of pleasure and interaction with the world. Activities such as sucking, biting, and chewing are not only essential for feeding but also serve as the infant’s primary means of exploring and understanding their environment [20].

In pediatric dentistry, Freud’s concept of the oral stage is particularly relevant. It suggests that early oral experiences play a crucial role in a child’s psychological development. Positive experiences during this stage, such as breastfeeding or being introduced to oral hygiene practices in a gentle and nurturing manner, can lead to a healthy relationship with oral care [19]. Conversely, negative experiences, such as pain or discomfort during teething or early dental visits, can lead to the development of anxiety or aversion toward oral care practices. Pediatric dentists, informed by these insights, often emphasize the importance of creating a positive first experience in the dental chair. By making the first dental visit pleasant and stress-free, dentists can help ensure that the child develops a positive association with oral health care, which can last into adulthood [21].

Freud’s insights on fear and repression

Freud’s exploration of anxiety and the mechanisms by which individuals cope with it, particularly in childhood, has provided valuable tools for understanding and managing dental anxiety in pediatric patients. Freud theorized that anxiety often stems from repressed fears and conflicts that reside in the unconscious mind. In children, these fears might manifest as a fear of the unknown, separation from parents, or fear of pain, all of which are common triggers of dental anxiety. Pediatric dentists today recognize that dental anxiety is not merely a reaction to an immediate threat but often a deep-seated fear that can be exacerbated by previous negative experiences or even by witnessing others in distress [22]. By understanding the roots of these fears, dentists can develop more empathetic and effective approaches to managing anxious children. Techniques such as desensitization, where children are gradually exposed to the dental environment in a non-threatening way, and the use of calming, child-friendly language are strategies that can help alleviate anxiety, making dental visits more manageable. Freud's concept of repression is also relevant when considering how children might deal with their dental fears. A child who represses their fear of the dentist may not express overt anxiety but could exhibit other behaviors, such as avoidance or acting out. Recognizing these signs allows pediatric dentists to address the underlying fear directly, rather than merely focusing on the overt behavior. This might involve using psychoanalytic-inspired techniques like play therapy or engaging in conversation to help the child express their fears in a safe and controlled environment [23].

The importance of early interventions and positive reinforcement

Freud’s emphasis on the significance of early experiences has led to a greater understanding of the need for early interventions in pediatric dentistry. Early dental visits, ideally starting at the eruption of the first tooth or by the child’s first birthday, are now recommended not only to monitor oral health but also to begin building a positive relationship between the child, their family, and the dental care team. During these early visits, pediatric dentists often employ techniques rooted in Freud’s understanding of early development [23,24]. They focus on making the experience enjoyable, using positive reinforcement, and providing parents with guidance on how to continue these positive practices at home. This might include demonstrating proper brushing techniques, discussing the importance of diet in preventing cavities, and encouraging parents to model good oral hygiene behaviors. Freud’s recognition of the role that parents play in a child’s early experiences also informs the way pediatric dentists engage with families. Dentists now understand that parental attitudes toward dentistry can significantly influence a child’s perception of dental care. By educating parents and involving them in the process, pediatric dentists can help ensure that children receive consistent messages about the importance of oral health, both in the dental office and at home [25,26].

## Conclusions

Beyond its roots in psychology, Sigmund Freud's groundbreaking contributions to psychoanalytic theory have had a profound impact on a wide range of academic fields. His ideas were novel at the time, but they are now fundamental to many academic programs, including those in dentistry and medicine. Specifically, the application of Freud's ideas has had a significant impact on pediatric dentistry, where it is essential to comprehend the psychological aspects of child behavior. Effective behavior control techniques for pediatric patients have been greatly influenced by Freud's understanding of the subconscious and early development.

Pediatric dentists now have useful tools at their disposal to address children's emotional and psychological needs because of the incorporation of psychoanalytic theory. This method has improved patient compliance and decreased anxiety by enabling more compassionate and knowledgeable care. The goals of pediatric dentistry, which aims to assist children's physical and emotional well-being, are in perfect harmony with Freud's emphasis on early experiences and their influence on behavior. Thus, Freud's influence continues to shape modern pediatric dentistry procedures in addition to serving as a pillar of psychoanalytic philosophy. His work is still enlightening and inspiring, revealing the eternal value of psychoanalytic concepts in comprehending and enhancing child behavior and care.
